# Effects of school-based high-intensity interval training on health-related fitness in adolescents

**DOI:** 10.3389/fphys.2024.1487572

**Published:** 2024-11-15

**Authors:** Rade Jovanović, Mladen Živković, Mima Stanković, Dajana Zoretić, Nebojša Trajković

**Affiliations:** ^1^ Faculty of Medicine, University of Niš, Physical Education Department, Niš, Serbia; ^2^ Faculty of Sport and Physical Education, University of Niš, Department of Theoretical-Methodological subjects, Niš, Serbia; ^3^ Faculty of Kinesiology, University of Zagreb, Zagreb, Croatia

**Keywords:** HIIT, youth, class, cardiorespiratory fitness, intervention

## Abstract

**Background:**

High-intensity interval training (HIIT) in school settings has been much less studied in adolescents. The aim of this study was to examine the impact of HIIT on health-related fitness in adolescents.

**Methods:**

The total sample consisted of 60 adolescents (age 16.33 ± 0.62 years) from secondary Grammar school, randomly divided into two groups: the experimental (EG) (30) and the control group (CG) (30). The experimental program (12-weeks; 2 times per week) involved two Tabata sessions during one physical education class lasting 4 min each. Participants were tested for health-related fitness components-cardiorespiratory fitness (The Shuttle Run Test (SRT) and strength, hand grip test, standing long jump (SLJ) and counter movement jump (CMJ).

**Results:**

Both the EG and the CG experienced significant positive changes in SRT (meters) and VO_2max_ values compared to baseline value (*p* < 0.05), however, the increase in the EG was significantly higher than that in the CG (SRT - η_p_^2^ = 0.111; VO_2max_ - η_p_^2^ = 0.111, *p* < 0.01). The EG showed significant improvement in SRT (meters) and VO_2max_ values compared to the CG (*p* < 0.01). Regarding the hand grip test results, a significant time × group interaction was found only for right hand (*p* < 0.01). Moreover, the improvements in SLJ and CMJ values was greater in EG than that in the CG group (SLJ- η_p_^2^ = 0.182; CMJ- η_p_^2^ = 0.112, *p* < 0.01).

**Conclusion:**

Findings indicate that HIIT implemented into physical education classes can result in significant improvements in selected health related fitness components in adolescents.

## 1 Introduction

Childhood and adolescence are key stages in the development and adoption of healthy habits. Insufficient physical activity (PA) (Kipping et al., 2008) is associated with all risks for developing cardiovascular diseases (Skinner et al., 2015; McMurray and Andersen, 2010), thus increasing the risk of premature mortality (Freedman et al., 2007; Franks et al., 2010). Furthermore, overweight and obesity, poor nutrition, reduced cardiorespiratory fitness, hypertension, chronic infections, and dyslipidaemia are evident in youth and become persistent health problems in adulthood (Simmonds et al., 2016).

Even though regular physical activity protects against the development of many diseases, studies showed that adolescents failed to meet the recommendations for physical activity in the past decade (Logan et al., 2014; Tapia-Serrano et al., 2022). Physical education (PE) classes often face structural limitations that hinder their ability to meet the recommended 60 min of moderate-to-vigorous physical activity (MVPA) per day for children and adolescents. As highlighted (Costigan et al., 2015b; Jurić et al., 2023), PE classes are typically constrained by limited time, with only a portion of the session dedicated to active movement, while the rest involves instruction, organization, or low-intensity activities. Moreover, Deutsch et al. (2022) emphasize that traditional warm-ups and activities in PE do not consistently engage students at the intensity levels required to promote cardiovascular fitness and muscular strength effectively. These limitations reduce opportunities for students to accumulate sufficient MVPA, underscoring the need for innovative approaches, which can maximize the use of available time through short bursts of high-intensity effort.

There is a strong reason to study how high-intensity interval training (HIIT) affects the quality of life and health of adolescents, as an important predictor of cardiometabolic health of the young (Segovia and Gutiérrez, 2020; Weston et al., 2020). Given that lack of time is cited as a major barrier for regular exercise, high intensity exercise of short duration is an excellent way to improve health in a short period of time (Costigan et al., 2015a). Moreover, it reduces the risk of cardiovascular diseases, in healthy and obese children and adolescents (Eddolls et al., 2017). Also, such a high intensity exercise in a short time with short rest intervals is a more suitable way of exercise for adolescents (Crisp et al., 2012; Buchan et al., 2013) However, in order to have an impact on cardiorespiratory fitness and health, it is recommended that an exercise program last a minimum of seven to 12 weeks (Steene Johannessen et al., 2013). Duncombe et al. (2022) showed in recent meta-analysis the effectiveness of HIIT training in school, aiming to promote the health of children and adolescents compared to a control group or another exercise modality,. The authors found significant improvements in anthropometric characteristics, cardiorespiratory fitness, in children and adolescents who practiced HIIT compared to the control group. However, Domaradzki et al. (2023) stated that the HIIT program introduced to a typical PE lesson can be considered partially effective and the programs should be designed specifically for males and females.

Tabata training has been considered one of the high-intensity “interval or intermittent” training (HIIT) methods (Tabata, 2019). It could vary considerably as regards of the characteristics of the training exercise, i.e., the exercise mode, intensity, and durations of exercise and rest. Weston et al. (2014) defined HIIT as “near maximal” effort performed at an intensity that elicits >80% (often 85%–95%) of the maximal heart rate. HIIT has been much less studied in children and adolescents in school settings, especially during the PE classes. Nevertheless, the results of HIIT studies in children and adolescent athletes have confirmed the improvement of cardiorespiratory fitness (shuttle run test - (SRT) (Buchheit et al., 2009), sprint (Sperlich et al., 2011), as well as 60 s sprint (Buchheit et al., 2010) and vertical jump (Tønnessen et al., 2011). The results of the study by Costigan et al. (2016) suggest that current physical activity and fitness levels among adolescents are low, which increases the risk of developing chronic diseases, and that it is necessary to include HIIT in mandatory Physical Education (PE) classes to improve cardiorespiratory function and body composition among adolescents. The results of several studies demonstrated improvement in health-related fitness in boys following HIIT (Meng et al., 2022; Corte de Araujo et al., 2012; Camacho-Cardenosa et al., 2016) Additionally, Petrušič et al. (2022) showed that only two additional training sessions a week are sufficient to bring about significant changes in physical fitness among adolescent girls.

Despite the growing body of literature examining the effects of HIIT on health-related fitness parameters across various age groups, there remains a notable gap in research specifically focusing on adolescents aged 15–17 years. While studies have investigated HIIT’s impact on adults and younger children, the unique physiological and develop mental characteristics of adolescents warrant dedicated attention. Understanding how HIIT influences health-related fitness components such as cardiovascular endurance and muscular strength in this age group is essential for designing effective and age appropriate exercise interventions. Moreover, there is a lack of studies investigating the effects of HIIT interventions as a part of physical education classes. Such research could contribute to evidence based strategies aimed at promoting optimal health and fitness during this critical period of physical development. Therefore, the aim of this study was to examine the effects of HIIT on health-related fitness in adolescents aged 15–17 years. It was hypothesized that the high-intensity program will contribute to better effects on the physical fitness of adolescents compared to the control group.

## 2 Materials and methods

### 2.1 Study design

The study employed a two-groups with pre post design, with participants divided into an experimental group and a control group. The intervention aimed to assess the impact of HIIT on health-related fitness (shuttle run performance, and strength outcomes, including standing long jump, countermovement jump (CMJ), and handgrip strength test) in adolescents. The experimental group replaced the traditional warm-up in physical education (PE) classes with a Tabata-based HIIT protocol, consisting of varied exercises performed twice a week for 12 weeks. Each session followed the standard Tabata format of 20 s of intense activity followed by 10 s of rest.

### 2.2 Participants

The total sample consisted of 60 adolescents (aged 15–17) from a secondary grammar school. Basic descriptive characteristics can be seen in Table 1. These participants were randomly assigned to two groups: an experimental group (EG) and a control group (CG), with 30 participants in each. The selection process involved adolescents from the first- and second-year classes (six classes in total) at the beginning of the school year. We used simple, non-returnable group randomization, conducted with the tool available at www.randomization.com, to ensure an unbiased allocation of participants. Biological maturity was calculated for each participant using the formula established by Mirwald et al. (2002). It is a non-invasive method that evaluates the time of greatest increase in height (PHV) taking into account anthropometric characteristics (body height, sitting height, leg length) and chronological age. The inclusion criteria for participation in the study were the following: male adolescents aged 15–17 years, not involved in any organized training processes beside school PE classes. At the time of the study, the participants had a medical certificate confirming they were healthy and able to meet the requirements expected of them. The exclusion criteria included adolescents with respiratory and cardiovascular diseases, developmental disabilities, chronic diseases, and those recovering from injuries or diseases, as well as active athletes. Prior to the onset of the study, the participants parents and the grammar school principal gave their written approval, which is in accordance with the Declaration of Helsinki. This research has been improved by the Ethical Committee, as the authors wrote in the end of the manuscript: Institutional Review Board Statement: The study was conducted in accordance with the Declaration of Helsinki, and results of this study resulted for the purposes of the doctoral dissertation, the topic of which was adopted at the University of Niš, Serbia (decision No. 8/18-01-007/23-031, approval date 06.11.2023). Furthermore, each parent gave written consent on behalf of their child to voluntarily participate in the study. The participants were allowed to withdraw from the experimental treatment at any time. In addition, the participants and their parents were acquainted with the importance and advantages of this study.

**TABLE 1:** Basic descriptive characteristics in the experimental and control group in the initial and final measurements.

Experimental group (n = 30)			Control group (n = 30)	
Outcome	Initial	Final	Initial	Final
BH (cm)	176.66 ± 8.77	177.26 ± 7.57	175.56 ± 5.71	176.66 ± 5.65
BM (kg)	66.73 ± 14.41	67.26 ± 13.38	71.09 ± 13.61	72.50 ± 13.63
BMI	21.51 ± 3.67	21.66 ± 3.41	23.01 ± 3.99	23.34 ± 3.97

Descriptive data are given as mean values ± standard deviations; *BH- body height, BM -body mass, BMI -body mass index*.

### 2.3 Testing procedures

Testing was conducted in two stages: the initial measurement, performed before the start of the experimental program, and the final measurement, conducted 12 weeks after the experimental program. Participants were tested for the basic anthropometric parameters and health related fitness components - cardiorespiratory fitness and strength. The measurements occurred in early October, following the start of the school year, and in late December. The testing as well as the experimental program were performed within a school athletic facility. For the purposes of all measurements and the experimental program, the same researchers were involved. Also, the measurements were carried out at the same time of day with the same schedule of tests for both measurements. Prior to the testing protocol, all participants underwent an introductory session where they were instructed on the correct form and technique for each fitness test. Since it is known that the type of warm-up can influence on performance (Patti et al., 2022), a standardized 15-min warm-up including dynamic excersises was administrated before testing.

In the morning, body composition was first measured, then tests of general and explosive strength, and finally, after a break, an aerobic endurance test, i.e. a shuttle run test.

#### 2.3.1 Body composition

Body height (BH) was measured with an anthropometer (SECA 214, Hamburg, Germany) according to standard procedures. The subject stands on a flat surface, with weight distributed equally on both legs. The shoulders are relaxed, the heels are gathered, and the head is placed in the position of the socalled. Frankfurt plane, which means that the imaginary line connecting the lower edge of the left orbit and the tragus helix of the left ear is horizontal. During the measurement, the subject rested his back on the anthropometer, maximally stretched.

Body mass (BM) - The Omron bf511 (Omron BF511, Kyoto, Japan) was used to obtain body mass values (Brtková et al., 2014).

Body mass index (BMI) - Height-weight indicator of an individual’s nutrition, which is valid for people over 20 years old and shows the ratio of body weight to height. According to the World Health Organization, a BMI less than 18.5 is considered underweight, while a BMI greater than 25 is considered overweight, and a value greater than 30 is considered obesity. It was obtained by formula (BMI = weight/height2).

#### 2.3.2 Hand grip test

This is a test used to measure the static strength of the hand flexor muscles. The Takei T.K.K.5401 GRIP-D (Takei Scientific Instruments Co., Ltd., Tokyo, Japan) hand grip dynamometer was used for all data collection. This was performed with the participants in standing, arm by their side with full elbow extension. The test taker has the right to two attempts, and the better one is entered. The hand on the dial must return to zero after the first attempt. The result of the test is the maximum muscle force of the hand grip Fmax expressed in kilograms (kg). The validity of this test was proved by Le-Ngoc and Janssen (2012).

#### 2.3.3 Standing long jump

The standing long jump test protocol involves the participant standing with feet shoulder width apart behind a marked line, bending the knees, and swinging the arms to generate momentum. The participants then jump forward as far as possible, landing on both feet. The distance from the starting line to the nearest point of contact on the landing (the back of the heels) is measured in cm. This process is repeated three times, with the longest jump recorded as the final result.

#### 2.3.4 Countermovement jump (CMJ)

The starting position of the participants is an upright position with a straight torso. The knee angle is 180°. The feet should be shoulder width apart. The participants should be in that position for at least 2 s. After that, they lower themselves until the knees are approximately bent at an angle of 90°. This is followed by a maximal effort, i.e., an explosive jump with legs extended and knees at an angle of 180° (Petrigna et al., 2019). Optojump (Optojump, Microgate, Bolzano, Italy) was used to assess the jump height, and the test result is the jump height expressed in cm. The test was repeated three times with a 30-s break between runs. The best achieved result was taken into further analysis. The validity of this test was proven by Markovic et al. (2004).

#### 2.3.5 Cardiorespiratory fitness

The Shuttle Run Test (SRT) also known as the 20-m shuttle run test or the beep test, is a commonly used assessment of cardiorespiratory fitness. It involves running back and forth continuously between two parallel 20 m lines. It consists of several stages (also called levels) each lasting about 1 min, with each stage consisting of several 20 m laps (also called shuttles). In each phase, the running speed is increased until the subject can no longer run 20 m in the given time with a sound signal (on two consecutive occasions) or when the subject stops due to fatigue. The result of this test is the maximum number of levels that participants complete before time runs out. The obtained result was used to calculate the maximum oxygen consumption according to the equation VO_2max_ = 31.025 + 3.238 X − 3.248A+ 0.1536 AX, in which X represents the speed in the last station in km/h and A the age. The required data (X) is read from existing tables. The validity of this test was proven by Matsuzaka et al. (2004).

### 2.4 Experimental program

Before the beginning of the experimental program, in agreement with the school director and professors, and with the written consent of the students parents, the participants were divided by randomly selection into two groups, experimental and control, with the included and excluded factors explained below. In addition to regular PE classes (45 min, twice a week), the experimental group underwent HIIT. The experimental part of the study was organized through a 12-week HIIT exercise program adapted to high school students and conducted in the preparatory phase of the class, twice a week, for a total duration of 9 min per class. First, all students did a 5 min exercise to warm up the muscles. In the preparatory phase of the class, the control group followed the PE lesson plan and program and traditional warm up, whereas the experimental group started with the Tabata protocol. A total of 8 exercises (running, burpees, split jump, jumping jack, push-ups, crunches, frog jump, Russian twist etc.) were performed within 4 minutes. The duration of the exercises was 20 s, with a break of 10 s, while at the end of an entire Tabata there was a break of 1 min. The exercise intensity was 80%–90% of the maximum heart rate (HRmax) as calculated by the shuttle run test (Ortega et al., 2023). The main and the final phase of the class were done together by the control and the experimental group following the PE lesson plan and program. The control group performed only their regular activities in PE classes.

### 2.5 Statistical analysis

We conducted an *a priori* power analysis using G*Power for a two-tailed t-test for dependent means (Faigenbaum and Myer, 2010). The input parameters included an effect size (d) of 0.55, an alpha level of 0.05, and a desired power (1-β) of 0.80. Based on these parameters, the total sample size required was 56 participants. Descriptive data are given as means standard deviations. Examination of the normality of the distribution of continuous variables, considering the size of the samples, was tested with the Kolmogorov-Smirnov test. The difference of the examined continuous variables within the examined groups, at the beginning and at the end of the experimental program, was determined by Paired samples t-tests in the case of normal distributions of the variables, or by the Wilcoxon Signed Ranks Test, in the case of deviations of the distribution’s variables than normal. The effects of high intensity interval training (HIIT) on changes in the fitness component was determined by a two-way analysis of variance (ANOVA) with repeated measures (group × time). The effect sizes were calculated using partial eta squared (η_p_^2^). The value *p* < 0.05 was used as the threshold of statistical significance.

## 3 Results

All data passed the normality tests. General descriptive parameters for both groups can be seen in Table 1. In terms of age, range of adolescents was 15–17 years, but the average age was 16.33 ± 0.62 (Y-PHV, i.e., years to and from peak height velocity = 0.1 ± 0.8). The t-test showed that there were no significant differences between groups in health-related fitness indices at baseline (all *p* > 0.05). A significant time × group interaction effects were found in all tested variables (*p* < 0.05) except for LHG where there was no significant interaction (*p* > 0.05). Comparing the SRT values both in meters and VO_2max_, it was determined that the values at the final measurement were statistically significantly higher compared to the initial measurement in both studied groups (Table 2; *p* < 0.05). However, the experimental group showed a significantly better increase in meters (SRT - η_p_^2^ = 0.111; *p* < 0.01) and in VO_2max_ performance (VO_2max_ - η_p_^2^ = 0.111, *p* < 0.01) after program (Table 2).

**TABLE 2:** Differences between experimental and control group in terms of Shuttle run test (SRT), hand grip test and explosive strength.

Experimental group (n = 30)				Control group (n = 30)			*F*	η_p_^2^
Outcome	Initial	Final	*p*	Initial	Final	*p*		
SRT (m)*	335.33 ± 199.39	457.33 ± 205.22	0.001	308.67 ± 117.99	348.00 ± 136.92	0.02	7.223	.111
SRT (VO2max)*	23.31 ± 2.52	25.79 ± 4.08	0.001	22.80 ± 2.52	23.63 ± 2.89	0.02	7.206	.111
RHG (kg)*	35.03 ± 7.19	36.80 ± 6.68	0.001	38.28 ± 9.09	38.93 ± 9.12	0.056	6.577	.102
LHG (kg)	33.45 ± 7.49	35.22 ± 7.15	0.01	35.60 ± 8.11	36.90 ± 8.10	0.09	.696	.012
SLJ (cm)*	175.73 ± 30.80	184.13 ± 26.89	0.001	182.10 ± 27.39	184.30 ± 26.41	0.83	12.931	.182
CMJ (cm)*	23.87 ± 6.92	25.94 ± 6.69	0.001	26.69 ± 4.26	27.36 ± 4.34	0.01	7.291	.112

Descriptive data are given as mean values ±SD; * signifficant difference between experimental and control group (*p* < 0.05); *SRT- shuttle run test*; *RHG − right hand grip dynamometer test, LHG − left hand grip dynamometer test*; SLJ, standing long jump; *CMJ, Countermovement jump;* η_p_^2^ *-partial eta squared*.

A significant group × time interaction was determined in right handgrip test (η_p_^2^ = 0.102; *p* = 0.02), with a significant increase (*p* = 0.001) in the EG but not in the CG (*p* = 0.56). The values of left handgrip test at the final measurement was higher in both groups compared to the initial measurement, but a statistically significant increase was found only in the experimental group (left hand −*p* < 0.01) (Table 2). Moreover, there was a significant group × time interaction for SLJ and CMJ. The improvements in SLJ and CMJ values was greater in EG than that in the CG group (SLJ- η_p_^2^ = 0.182; CMJ- η_p_^2^ = 0.112, *p* < 0.01).

## 4 Discussion

The aim of this study was to examine the impact of HIIT on health-related fitness in adolescents aged 15–17 years. The main results of this study were that the experimental group showed a significant improvement in cardiorespiratory fitness and strength compared to control group after implementing HIIT as a substitute for traditional warm-ups during the PE classes over the school year. Specifically, HIIT induced significant improvement of SRT in meters and VO_2max_ compared to the control group. Additionally, the values of hand grip test as well as the values of standing long jump and CMJ were significantly higher compared to the control group. Having in mind there were obvious improvements in experimental group compared to the control group, the hypothesis was accepted.

The PA and fitness levels among adolescents are low during past decades (Costigan et al., 2016; Nevill et al., 2023; Aubert et al., 2021). In addition, recent review showed that most adolescents failed to meet the 24-Hour Movement Guidelines, which also include the 60 min of moderate to vigorous physical activity daily (Tapia-Serrano et al., 2022). HIIT training was proposed as intervention designed to increase moderate-to-vigorous PA in Physical Education classes (Muntaner-Mas and Palou, 2017). This research results are in line with above mentioned proposal. The HIIT implemented during mandatory PE classes, improved health related fitness in healthy adolescents.

Specifically, the 12-week HIIT program of this study showed better SLJ and CMJ results in the experimental group compared to the control group, which is in agreement with the results of several recent studies (da Silva et al., 2020; Li et al., 2023; Abassi et al., 2023). For example, Costigan et al. (2015a) highlight significant improvements in vertical jump performance following a school-based HIIT intervention, emphasizing the program’s feasibility. Similarly, Jurić et al. (2023) found that incorporating HIIT in physical education classes not only improved general fitness but also specifically enhanced standing long jump outcomes, suggesting the effectiveness of such protocols in fostering explosive leg power. These findings align with the results of Cvetković et al. (2018), who reported that a 12-week HIIT program led to substantial gains in both vertical jump and standing long jump, indicating that these activities can effectively promote explosive power among children and adolescents. Similar results for standing long jump and vertical jump (*p* < 0.05) were found following running-based and body-weight based HIIT in healthy adolescents (Li et al., 2023). Together, these studies demonstrate the potential of HIIT interventions to improve physical fitness outcomes, particularly those requiring lower-body explosive strength, when embedded within the school curriculum. On the other hand, some studies showed no significant improvement, especially for the vertical jump. After a 12-week school intervention based on small sided games (the program included futsal, basketball, handball, and volleyball), Petrušić et al. (2022) found no significant improvement for CMJ. Similar results were found by Trajković et al. (2020) after a small-sided games football intervention at school, which also showed small effects (3.5%). In contrast, recreational football training in obese school children showed significantly greater effects on CMJ (17.0%) (Cvetković et al., 2018). Differences in intervention duration among different studies, as well as differences in weight status and other characteristics, could be a possible explanation for this discrepancy in results between studies. In addition, different protocols might have been used to test vertical jump height, which could also add to the differences in results.

García-Hermoso et al. (2020) stated that early intervention that target CRF in children may be related with maintaining health parameters later in life. Recent systematic review and meta-analysis conducted by Martin-Smith et al. (2020) showed that HIIT is a sustainable and effective method for improving cardiorespiratory fitness in adolescents. The results of the current study confirm these conclusions, displaying significant improvements in the 20-m shuttle run test following 12 weeks of Tabata protocol. Studies such as Jurić et al. (2023) and Li et al. (2023) reported also a significant improvements in VO_2max_ and overall aerobic capacity among participants who engaged in HIIT programs during physical education classes. These interventions, typically characterized by alternating high-intensity efforts with recovery periods, efficiently stimulate cardiovascular adaptations, even within relatively short timeframes. Similarly, González-Gálvez et al. (2024) found that HIIT protocols improved cardiorespiratory markers and metabolic health in adolescents. Furthermore, Popowczak et al. (2022) found that a 10 weeks HIIT Tabata exercise program in adolescents improved CRF, which is in agreement with the results of this study. However, some studies have also observed no significant improvements in cardiorespiratory fitness following HIIT interventions. Costigan et al. (2015b) noted variability in results, with some participants showing limited aerobic gains. Similarly, to our study, Alonso-Fernández et al. (2019) implementing the HIIT protocol in PE classes, did not find a significant difference compared to the control group in healthy adolescents. However, the author stated that findings suggest a promising outlook on how incorporating HIIT as a substitute for traditional warm-ups can improve students’ physical fitness through the protocol of functional bodyweight exercises, all without impacting the remaining curriculum required in PE classes over the school year. Possible reasons for the lack of improvement include inadequate training intensity or volume, individual differences in baseline fitness levels, varying levels of motivation among participants, and the challenge of maintaining consistent effort throughout the sessions. Additionally, factors such as insufficient recovery between sessions and external lifestyle factors (e.g., diet, sleep) may influence the overall effectiveness of HIIT programs in improving aerobic fitness.

It is well documented that greater grip strength is related to longitudinal health maintenance and health improvements in adolescents (Peterson et al., 2018). Although HIIT is primarily designed to improve cardiovascular fitness, some studies have reported positive effects on muscular strength, including handgrip strength. For example, González-Gálvez et al. (2024) found improvements in upper-body strength, including handgrip, following a structured HIIT program, suggesting that high-intensity efforts can stimulate neuromuscular adaptations. Similarly, Jurić et al. (2023) observed that participation in HIIT during physical education classes positively influenced muscle strength, possibly due to the inclusion of bodyweight exercises and functional movements within the training protocol.

However, not all studies have shown significant improvements in handgrip strength. Costigan et al. (2015b) reported limited gains in muscular strength, including handgrip, which may reflect the nature of HIIT programs that focus more on cardiovascular demands rather than maximal strength development. The present results support the above mentioned inconsistent results for grip strength, with significant differences between groups only for right hand handgrip test. Reasons for improvement could include the indirect activation of upper-body muscles during explosive movements and improved neuromuscular coordination. On the other hand, the absence of improvement might result from insufficient specific strength training, low engagement of the upper limbs in HIIT exercises, or the relatively short duration of the intervention, which may not be enough to develop maximal strength in young populations.

Some possible limitations of the study should be mentioned. First of all, the number of participants is limited, and some participants declined to participate, which could introduce selection bias into the study’s findings. Larger sample sizes from school-based research are required in the future to confirm HIIT’s usefulness as an intervention for adolescents. Second, because this study only involved males, we were unable to determine gender differences and the effects of exercise intervention. The absence of a objectively measured physical activity and strictly controlled diet may cause may have influenced the results. However, all participants were adwised to continue with their usual diet and they were not engaged in organized sport programs outside the school PE class. Nevertheless, a key strength of the study is that HIIT was implemented in a school setting during PE class warm-ups, ensuring feasibility, practicality, and seamless integration into students’ existing routines.

## 5 Conclusion

Findings from the present study indicate that HIIT training can result in significant improvements in selected health related fitness components (SRT, SRTVO2max, RHG, LHG and CMJ) in adolescents. Having in mind that the intervention was formed as a part of the PE class, in which only the warm up phase (HIIT or traditional) differed, the results make a promising strategy for combating sedentary lifestyles in adolescents. Moreover, if we exert influence on adolescents to adopt physical activity as a daily routine, it will undoubtedly have a significant impact on reducing the risk of developing bad habits and a sedentary lifestyle in adulthood.

## Data Availability

The original contributions presented in the study are included in the article/supplementary material, further inquiries can be directed to the corresponding author.
